# Supplementary material for: Saccharomyces boulardii (CNCM I-745) improves intestinal damage in sepsis by remodeling intestinal flora structure
Source: Front Cell Infect Microbiol. 2025 Jul 31;15:1602792. doi: 10.3389/fcimb.2025.1602792 (PMC12350329; doi:10.3389/fcimb.2025.1602792)
Supplement: Supplementary file 1 [file DataSheet1.zip › summary/summary/6_Taxonomy_community/1_Taxonomy_profiling/5_Family/Group/Family_abund_top30.Group.cluster.pdf]

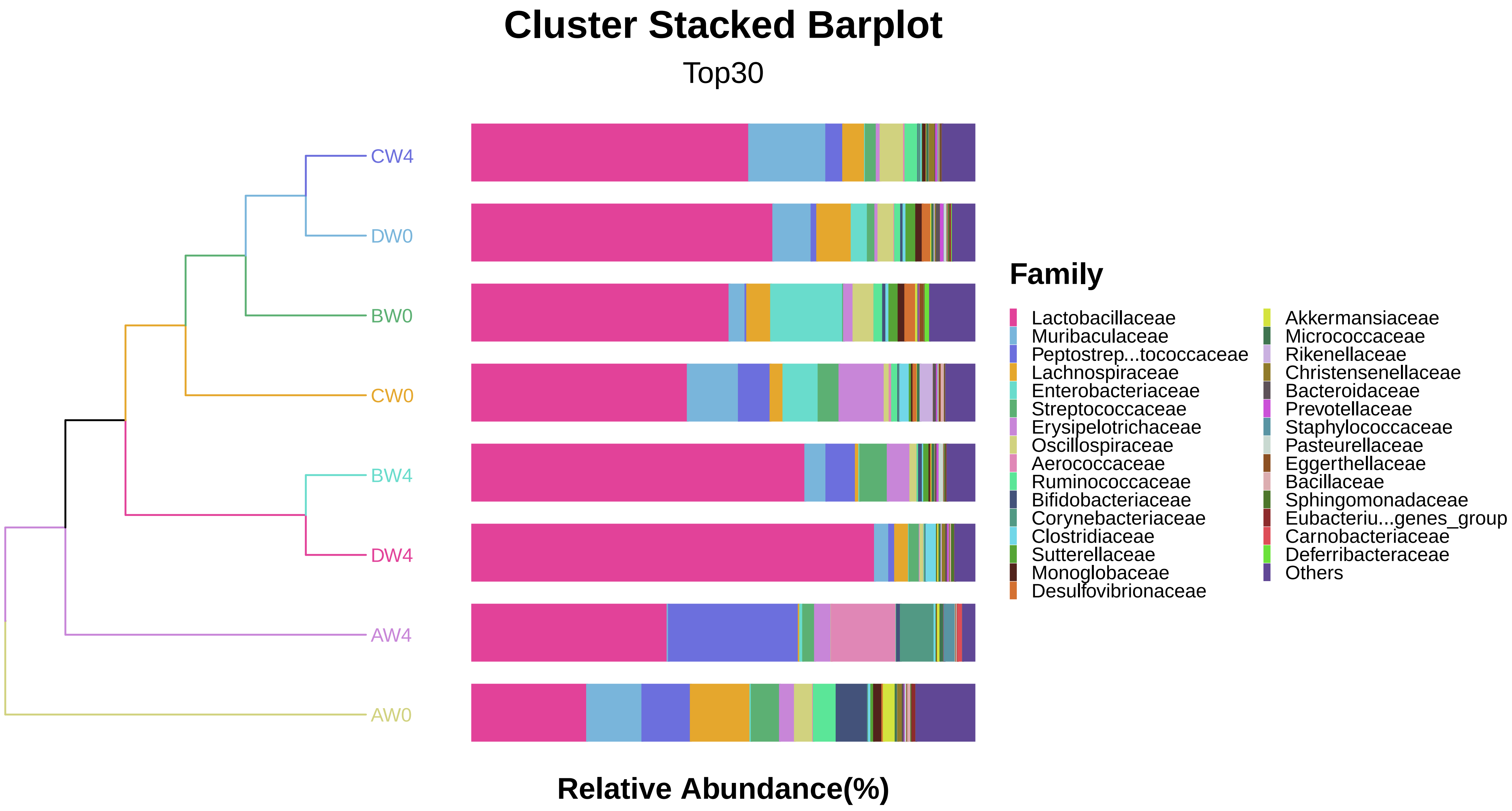
Cluster Stacked Barplot
Top30
CW4
DW0
BW0
CW0
BW4
DW4
AW4
AW0
Relative Abundance(%)
Family
Lactobacillaceae
Muribaculaceae
Peptostrep...tococcaceae
Lachnospiraceae
Enterobacteriaceae
Streptococcaceae
Erysipelotrichaceae
Oscillospiraceae
Aerococcaceae
Ruminococcaceae
Bifidobacteriaceae
Corynebacteriaceae
Clostridiaceae
Sutterellaceae
Monoglobaceae
Desulfovibrionaceae
Akkermansiaceae
Micrococcaceae
Rikenellaceae
Christensenellaceae
Bacteroidaceae
Prevotellaceae
Staphylococcaceae
Pasteurellaceae
Eggerthellaceae
Bacillaceae
Sphingomonadaceae
Eubacteriu...genes_group
Carnobacteriaceae
Deferribacteraceae
Others
